# Single human oocyte transcriptome analysis reveals distinct maturation stage‐dependent pathways impacted by age

**DOI:** 10.1111/acel.13360

**Published:** 2021-04-28

**Authors:** Sílvia Llonch, Montserrat Barragán, Paula Nieto, Anna Mallol, Marc Elosua‐Bayes, Patricia Lorden, Sara Ruiz, Filippo Zambelli, Holger Heyn, Rita Vassena, Bernhard Payer

**Affiliations:** ^1^ Centre for Genomic Regulation (CRG) The Barcelona Institute of Science and Technology Barcelona Spain; ^2^ Clínica EUGIN Barcelona Spain; ^3^ CNAG‐CRG Centre for Genomic Regulation (CRG) The Barcelona Institute of Science and Technology Barcelona Spain; ^4^ Universitat Pompeu Fabra (UPF) Barcelona Spain

**Keywords:** advanced maternal age, ageing, assisted reproduction, BMI, fertility, human oocyte, single‐cell RNA‐Seq, transcriptomics

## Abstract

Female fertility is inversely correlated with maternal age due to a depletion of the oocyte pool and a reduction in oocyte developmental competence. Few studies have addressed the effect of maternal age on the human mature oocyte (MII) transcriptome, which is established during oocyte growth and maturation, however, the pathways involved remain unclear. Here, we characterize and compare the transcriptomes of a large cohort of fully grown germinal vesicle stage (GV) and in vitro matured (IVM‐MII) oocytes from women of varying reproductive age. First, we identified two clusters of cells reflecting the oocyte maturation stage (GV and IVM‐MII) with 4445 and 324 putative marker genes, respectively. Furthermore, we identified genes for which transcript representation either progressively increased or decreased with age. Our results indicate that the transcriptome is more affected by age in IVM‐MII oocytes (1219 genes) than in GV oocytes (596 genes). In particular, we found that transcripts of genes involved in chromosome segregation and RNA splicing significantly increased representation with age, while genes related to mitochondrial activity showed a lower representation. Gene regulatory network analysis facilitated the identification of potential upstream master regulators of the genes involved in those biological functions. Our analysis suggests that advanced maternal age does not globally affect the oocyte transcriptome at GV or IVM‐MII stages. Nonetheless, hundreds of genes displayed altered transcript representation, particularly in IVM‐MII oocytes, which might contribute to the age‐related quality decline in human oocytes.

## Graphical abstract

Here we used single‐oocyte RNA‐Seq to study the transcriptome changes occurring during human oocyte aging. We sampled Germinal Vesicle (GV) stage oocytes from women of age 18–43 and analyzed them directly (GV) or after in vitro maturation (IVM‐MII). We observed that age has a bigger impact on the IVM‐MII transcriptome. Transcripts belonging to biological pathways critical for oocyte maturation and function either increased (green, top) or decreased (red, bottom) in representation with age.

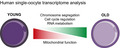

## 1 INTRODUCTION

Throughout recent decades, maternal age at the birth of the first child has significantly increased (Matthews & Hamilton, 2009). Consequently, assisted reproductive techniques are used with increasing frequency in women older than 35, as natural fertility decreases significantly beyond this age (Howles et al., 2006). There are two reasons for this decline in fertility; the depletion of the oocyte pool over time and a reduction in oocyte quality, which leads to increased incidence of aneuploidies, lower embryo developmental rates and increased pregnancy loss (Nagaoka et al., 2012).

During foetal development, oocytes initiate meiosis and arrest at the diplotene stage of prophase I. At this stage, oocytes present a characteristic nucleus called germinal vesicle (GV) and remain quiescent for several years. Subsequently, throughout a woman's reproductive lifespan, oocyte growth, and maturation are triggered. On a monthly basis, in response to a luteinizing hormone (LH) surge, the dominant oocyte will resume meiosis reaching the nuclear maturation stage of metaphase II (MII) and be ovulated. At the end of oogenesis, it is imperative for the oocyte to have reached the cytoplasmic and nuclear maturity required to support embryonic development upon fertilization. Several factors are known to impair oocyte quality and thus their developmental potential, including age and body mass index (BMI).

Many biological pathways have been postulated to be responsible for the age‐related oocyte quality drop (Nagaoka et al., 2012); for example, mitochondria‐related defects (Almansa‐Ordonez et al., 2020) and epigenetic changes affecting gene expression (Chamani & Keefe, 2019). In addition, another very important factor is the increased incidence of embryo aneuploidies with maternal age. During meiotic arrest, the linkage between chromatids is maintained by crossovers and proteins such as cohesins. Studies in mouse and human suggest that age‐related chromosome segregation errors could be due to gradual loss of cohesion and kinetochore compaction (Burkhardt et al., 2016; Gruhn et al., 2019; Smoak et al., 2016; Zielinska et al., 2019) and altered microtubule dynamics, which lead to aberrant spindle assembly and therefore to non‐disjunction events (Nakagawa & FitzHarris, 2017). In mice, it has been shown that cohesin and centromeric proteins are incorporated into meiotically arrested chromosomes early during development and remain there without significant turnover until oocyte maturation much later in life (Burkhardt et al., 2016; Smoak et al., 2016). However, in oocytes of other species like starfish, centromeric nucleosomes are continuously replenished during meiotic arrest (Swartz et al., 2019). Therefore, it remains unclear to which degree proteins important for chromosome cohesion and centromere identity are turned over in human oocytes, which can remain in meiotic arrest for decades.

In addition to age, an abnormal BMI also has an effect on oocyte quality. This is reflected by the number of oocytes retrieved, fertilization rate, embryo quality, pregnancy rate and miscarriage percentage (Brower et al., 2013; Machtinger et al., 2012; Shah et al., 2011). Similarly to aged oocytes, Machtinger et al. observed that oocytes from obese women presented aberrant spindles and misalignment of chromosomes. However, some discrepancies exist among published data and the underlying biological relation between BMI and impaired capacity of reproduction is still missing. A deeper understanding of the mechanisms driving the decline of oocyte quality with age and abnormal BMI is therefore needed. In particular, it remains elusive to which extent the transcriptome plays a role in human oocyte ageing and BMI‐related infertility.

Several studies have been performed in human oocytes using microarray analysis (reviewed in Labrecque & Sirard, 2014). Single‐cell RNA sequencing (scRNA‐seq) techniques developed over the last decade are among the leading tools for exploring tissue heterogeneity at a cellular level (Svensson et al., 2018). These techniques facilitate the identification of transcriptional differences between cells that would remain undetectable with conventional bulk RNA sequencing. More recent studies have therefore applied scRNA‐seq to analyse age‐related differences in the transcriptome of MII oocytes or the impact of in vitro maturation in GV oocytes from young vs. advanced maternal age women, by analysing the transcriptome in GVs and IVM‐MII oocytes (Reyes et al., 2017; Zhang et al., 2020). The main pitfall has been the low number of oocytes used in these studies.

Here, we apply scRNA‐seq analysis on the poly(A)‐RNA transcriptome of a large number of (*n* = 72) single human GV oocytes obtained during ovum pick‐up after ovarian stimulation of women ranging from 18 to 43 years of age. Additionally, applying an experimental in vitro maturation protocol towards the MII stage, we identified differences in the representation of RNAs related to specific biological processes (chromosome segregation, cell cycle regulation, mitochondrial function and RNA metabolism) that were correlated with women's age or BMI. Furthermore, we found, through network analysis, potential master regulators involved in reproductive ageing. Therefore, our data suggest that RNA‐turnover might play an instructive role in oocyte ageing thereby advancing our understanding of the reproductive ageing process.

## 2 RESULTS

**FIGURE 1:**
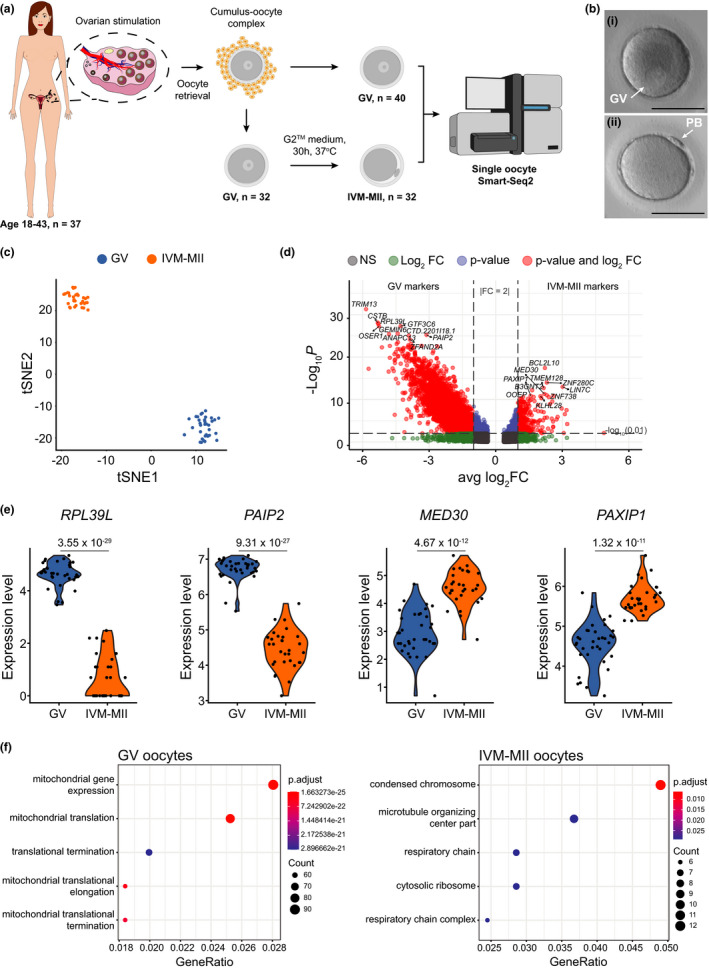
Single‐cell transcriptome profiling of human oocytes. (a) Schematic representation of the experimental design. Briefly, 37 women were recruited. The mean woman age was 28.8 years (*SD* = 7.7, range 18–43), and from each woman we included between 1–4 GV oocytes. GV oocytes were analysed directly as GV (*n* = 40) right after denudation or, after 30 h in G2^TM^ medium, as in vitro matured metaphase II (IVM‐MII, *n* = 32) oocytes. Their transcriptome was compared by single‐cell RNA‐sequencing analysis (Smart‐seq2). (b) Exemplary pictures of a germinal vesicle (i) and a IVM‐MII (ii) oocyte included in the study. Scale bar = 100 µm. (c) Oocytes cluster according to their maturation stage. (d) Differentially expressed genes between the GV and the IVM‐MII groups are represented in red. Labels correspond to the top10 differentially expressed genes in each category after filtering for fold change 2 (avg_log2FC > 1) and sorting markers according to their *p*‐value (cutoff = 0.01). Total number of variables: 12,431. (e) Example of two GV markers (*RPL39L*: Ribosomal Protein L39‐Like; *PAIP2*: Poly(A) binding protein Interacting Protein 2) and two IVM‐MII markers (*MED30*: MEDiator complex subunit 30; *PAXIP1*: PAX Interacting Protein 1). (f) Gene Ontology enrichment analysis of each maturation stage. The top 5 activated GO terms are shown. *p*‐values were adjusted using the FDR method. GeneRatio: number of genes related to the GO term/total number of significant genes. GV: Germinal Vesicle; IVM‐MII: in vitro matured metaphase II, PB: Polar Body

The main goal of this study was to investigate changes in transcriptome associated with oocyte ageing. For that purpose, we collected GV oocytes from 37 women within an age range of 18–43 years and either subjected them directly at the GV stage (*n* = 40), or after in vitro maturation to IVM‐MII stage (*n* = 32), to single‐oocyte RNA sequencing using the Smart‐seq2 protocol (Picelli et al., 2013) (Figure 1a,b, Table S1, Figures S1 and S2).

### 2.1 Oocytes cluster according to maturation stage

Firstly, we aimed to elucidate which parameter had the biggest impact on the oocyte transcriptome when performing unbiased clustering of our single oocyte expression data. Dimensionality reduction analysis by tSNE along with graph‐based clustering using the Louvain algorithm revealed two groups of oocytes, with maturation stage being the differentiating feature between clusters (Figure 1c). In order to identify transcripts specifically enriched at each maturation stage, we considered those genes with a fold change (FC) > |2| and *p*‐value < 0.01 as differentially expressed genes (DEGs). Thereby, we found 4445 transcripts overrepresented in GV oocytes (out of 11,603 ± 373 SEM detected genes in GVs) and 324 transcripts overrepresented in IVM‐MII oocytes (out of 8586 ± 435 SEM detected genes in IVM‐MIIs) (Figure 1d,e, Table S2, Figure S3). The top 10 genes (according to *p*‐value) identified to be differentially represented in GV or IVM‐MII oocytes are listed in Table 1. Interestingly, of the genes we identified as overrepresented in each maturation stage at the RNA level, some have also been previously found to be stage‐specific GV (TDRKH) and MII (WEE2, DNMT1) protein markers by single‐cell proteomics (Figure S3) (Virant‐Klun et al., 2016). This suggests that the differences we observed in RNA representation are also reflected at the protein level for these mentioned genes. Deeper proteomic investigations would be required to see whether this is also the case for the other overrepresented transcripts in each maturation stage.

**TABLE 1:** Top 10 genes identified as GV or MII markers, ranked according to *p*‐values

Gene name	Description	avg_log_2_FC	*p*‐value
Top 10 transcripts overrepresented in GVs			
TRIM13	Tripartite Motif Containing 13	5.846389779	1.67E−32
CSTB	Cystatin B	5.317731081	2.85E−29
RPL39L	Ribosomal protein L39 Like	5.294740083	3.55E−29
GTF3C6	General Transcription Factor IIIC Subunit 6	4.286292808	1.81E−28
OSER1	Oxidative Stress Responsive Serine Rich 1	5.256582491	2.14E−28
PAIP2	Poly(A) Binding Protein Interacting Protein 2	3.120456569	9.31E−27
GEMIN6	Gem Nuclear Organelle Associated Protein 6	4.417622279	1.01E−26
ANAPC13	Anaphase Promoting Complex Subunit 13	4.807780144	1.64E−26
CTD.2201l18.1		3.913549439	2.76E−26
ZFAND2A	Zinc Finger AN1‐Type Containing 2A	3.868706352	2.73E−25
Top 10 transcripts overrepresented in IVM‐MIIs			
BCL2L10	BCL2 Like 10, apoptosis regulator	2.217812201	3.76E−18
ZNF280C	Zinc Finger Protein 280C	2.303678722	9.87E−15
LIN7C	Lin‐7 Homolog C, Crumbs Cell Polarity Complex Component	3.011566409	1.04E−13
TMEM128	Transmembrane Protein 128	2.162524441	1.94E−13
B3GNT2	UDP‐GlcNAc:BetaGal Beta‐1,3‐N‐Acetylglucosaminyltransferase 2	1.640027602	6.51E−13
MED30	Mediator Complex Subunit 30	2.191037289	4.67E−12
OOEP	Oocyte Expressed Protein	1.233834045	2.48E−11
PAXIP1	PAX Interacting Protein 1	1.573832651	1.32E−11
ZNF738	Zinc Finger Protein 738	2.300681536	9.24E−12
KLHL28	Kelch Like Family Member 28	2.017362891	1.64E−11

Note

The rest of maturation stage markers and their corresponding *p*‐values can be found in Table S2.

We then performed gene ontology (GO) term enrichment analysis on the overrepresented genes to compare the transcriptomes between GV stage and IVM‐MII stage (Figure 1f). Within the list of genes with increased transcript representation in GV oocytes, we found GO terms related to mitochondrial gene expression (e.g. many genes constituting the large and small mitochondrial ribosomes such as *MRPL27* and *MRPS22*). On the other hand, genes with a higher transcript representation in IVM‐MII oocytes belonged to GO terms related to chromosome condensation (e.g. CENPK, ADD3) and microtubule‐organizing centre (e.g. CETN3, KIF3A) (Table S3).

In summary, unsupervised clustering of our data identified maturation stage as the main differentiator between oocytes. We identified DEGs for GV and IVM‐MII oocytes and revealed GO pathways enriched for each stage.

### 2.2 Transcript representation changes with age for specific gene groups

**FIGURE 2:**
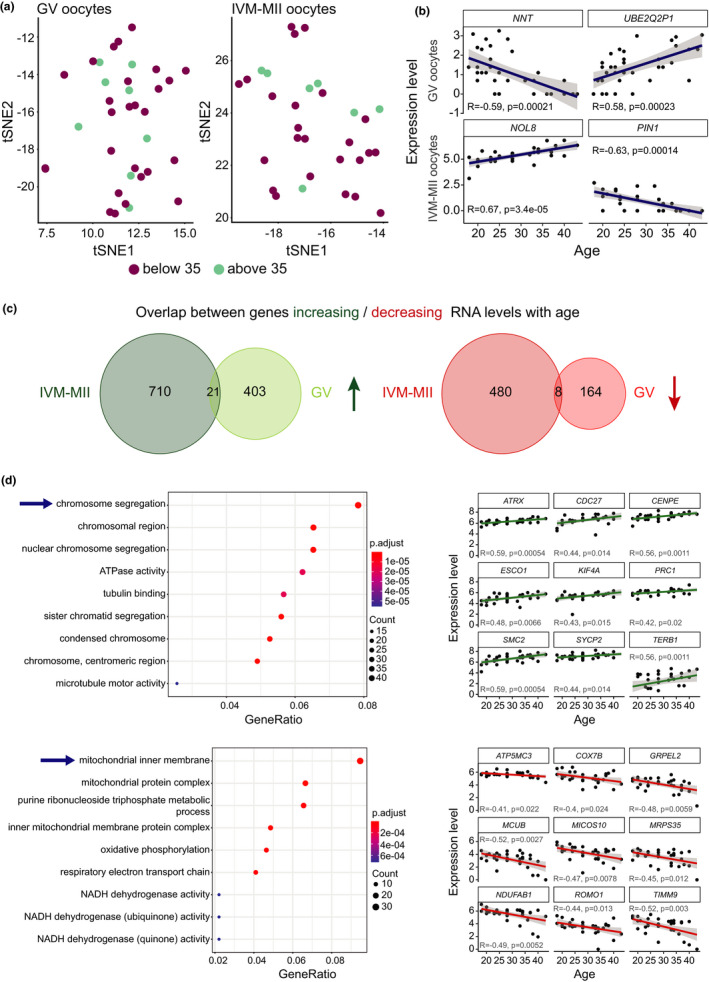
Analysis of gene expression correlation with age. (a) Cluster analysis of oocytes at each maturation stage, GV and IVM‐MII, independently. (b) Examples of genes that correlate with age. *NNT* and *UBE2Q2P1* are genes found in the GV set of oocytes, while *NOL8* and *PIN1* were identified within the IVM‐MII oocyte population. (c) Venn diagrams showing the overlap between genes changing RNA levels with age in GV and IVM‐MII oocytes. In green, genes whose RNAs abundance increased with age, in red the number of genes whose RNAs abundance decreased with age. (d) Gene ontology analysis of genes that change with age in IVM‐MII oocytes (left) and examples of how expression levels of genes in some of the most significant GO terms (indicated by arrows) vary with age (right). Upper panel: genes belonging to the GO term ‘chromosome segregation’ for which expression increases with age. Lower panel: genes belonging to the GO term ‘mitochondrial inner membrane’ for which expression decreases with age

With maturation stage being the main variable distinguishing our two cell clusters (Figure 1c), we decided to perform dimensionality reduction analysis by tSNE and clustering on each one (GV and IVM‐MII) separately to determine whether age (i.e. <35 vs. >35 years) could be a differentiating feature within each maturation stage. However, this analysis did not reveal separate age‐related clusters within GV oocytes or IVM‐MII oocytes (Figure 2a). Furthermore, neither the number of poly(A)‐RNA molecules nor of expressed genes detected per oocyte significantly changed with age (Figure S2), providing additional evidence that the oocyte transcriptomes did not change globally with age.

Next, as oocyte quality declines with age, we sought to identify specific genes, of which RNA representation would increase or decrease in an age‐dependent manner. Therefore, we performed correlation tests between gene expression and age independently for GV and IVM‐MII oocytes. For each detected gene, we obtained a Pearson correlation value (*R*) and a *p*‐value. The genes that presented an absolute correlation value equal or over 0.3 (*R* ≥ |0.3|) and a *p*‐value below 0.05 were considered as genes for which transcript representation correlated either positively (increased) or negatively (decreased) with age (Table S4). Following these criteria, we identified a total of 596 genes with altered RNA representation during ageing within the GV population and 1219 genes within the IVM‐MII population. In Table 2, the top 5 genes with increased/decreased RNA representation within the GV/IVM‐MII population are listed. In Figure 2b, we show an example of each category.

**TABLE 2:** Top 5 genes (according to *R*‐value) with increased/decreased RNA representation in function of age identified within the GV/IVM‐MII population independently

Gene name	Description	*R* value	*p*‐value
Top 5 GV up			
ENSG00000278292	Ret Finger Protein Like 4A Pseudogene 6	0.6294745	5.09827E−05
UBE2Q2P1	Ubiquitin Conjugation Enzyme E2 Q2 Pseudogene 1	0.58455502	0.000226527
WFIKKN2	WAP, Follistatin/Kazal, Immunoglobulin, Kunitz And Netrin Domain Containing 2	0.58369917	0.000232566
LINC02022	Long Intergenic Non‐Protein Coding RNA 2022	0.57281807	0.000322912
ENSG00000227240	lncRNA, new transcript	0.000322912	0.000339781
Top 5 GV down			
NNT	Nicotinamide Nucleotide Transhydrogenase	−0.5872276	0.000208561
DCK	Deoxycytidine Kinase	−0.5281484	0.001110088
KAT8	Lysine Acetyltransferase 8	−0.5205088	0.001348543
RGS18	Regulator Of G Protein Signaling 18	−0.4935282	0.002589092
ARHGEF26	Rho Guanine Nucleotide Exchange Factor 26	−0.4934728	0.002592426
Top 5 IVM‐MII up			
NOL8	Nucleolar Protein 8	0.672405618	3.42412E−05
TNIK	TRAF2 And NCK Interacting Kinase	0.661684117	5.04619E−05
ESCO2	Establishment Of Sister Chromatid Cohesion N‐Acetyltransferase 2	0.650871928	7.3487E−05
AFDN	Afadin, Adherens Junction Formation Factor	0.638270876	0.000111848
TPR	Translocated Promoter Region, Nuclear Basket Protein	0.634740326	0.0001254
Top 5 IVM‐MII down			
PIN1	Peptidylprolyl Cis/Trans Isomerase, NIMA‐Interacting 1	−0.632135194	0.00013632
SERF2	Small EDRK‐Rich Factor 2	−0.621504263	0.000190188
C12orf75	Chromosome 12 Open Reading Frame 75	−0.620631179	0.000195356
TLR5	Toll Like Receptor 5	−0.605605391	0.000306149
KIAA1671	Uncharacterized Protein KIAA1671	−0.588558598	0.000496349

Gene name	Description	*R* value		*p*‐value	
		GV	IVM‐MII	GV	IVM‐MII
Examples of genes with increased transcript representation with age					
FAM201B	Family With Sequence Similarity 210 Member B	0.36	0.44	0.03	0.01
TERB1	Telomere Repeat Binding Bouquet Formation Protein 1	0.36	0.38	0.03	0.03
RFC1	Replication Factor C Subunit 1	0.42	0.45	0.01	0.01
Examples of genes with decreased transcript representation with age					
ND1	Mitochondrially Encoded NADH: Ubiquinone Oxidoreductase Core Subunit 1	−0.35	−0.47	0.04	0.01

Note

The rest of genes, together with the *R* and *p*‐values can be found in Table S4.

We then created Venn diagrams in order to visualize the degree to which the genes whose RNA representation positively or negatively correlated with age overlapped between GV and IVM‐MII oocytes (Figure 2c). To our surprise, we found very little overlap between the 424 genes in GV oocytes and the 731 genes in IVM‐MII oocytes whose RNA representation increased with age, with only 21 genes increasing both in GV and IVM‐MII oocytes. Likewise, from the 172 and 488 genes with age‐related decreased transcript representation in GV and IVM‐MII, respectively, only 8 overlapped. This suggests that age affects the transcript representation predominantly of different genes in GV and IVM‐MII oocytes. Among the few genes whose RNA representation increased with age in both GV and IVM‐MII oocytes, we found *FAM210B*, a mitochondrial factor that has been associated with human ovarian cancer (Sun et al., 2017). Further examples were *TERB1*, which encodes a meiosis‐specific telomere‐associated protein involved in attaching the meiotic telomere to the inner nuclear membrane, and *RFC1*, encoding a subunit of the replication factor C, a DNA polymerase accessory protein required for DNA replication and repair and that might also play a role in telomere stability. None of these three genes varied significantly in RNA representation between GV and IVM‐MII stage, suggesting that their age‐related increase in RNA levels was already present at the GV stage and was maintained through the in vitro maturation step to the IVM‐MII stage. Among the genes whose RNA levels decreased with age in both GV and IVM‐MII stages, we found *ND1*, which is involved in electron transport in the mitochondrial respiratory chain.

After this preliminary analysis, the genes present in the correlation lists (Table S4) were further analysed using clusterProfiler (Yu et al., 2012). We looked for enriched GO terms in the lists of genes whose transcript representation increased with age separately from those whose transcript representation decreased with age. When analysing the GV stage oocytes we did not find any particular GO term significantly enriched (*p* < 0.05) after adjusting the *p*‐values (FDR method), neither in the set of genes increasing in RNA representation with age nor in the ones decreasing. In contrast, in the GO term enrichment analysis performed on genes whose transcript representation changed with age on the IVM‐MII stage oocytes, we did find significantly enriched GO terms (FDR, *p* < 0.05) (Figure 2d, left panels). The most enriched GO terms we found within the genes that increased in transcript representation with age were predominantly related to chromosome segregation. To name some key examples, the cohesin loading and release factors NIPBL and WAPL, the cohesin SMC3, the condensin SMC4, the double‐strand break repair factor SMC5, the synaptonemal complex member SYCP2 and the centromeric proteins CENPC, CENPE, CENPF, CENPM and INCENP were all increased at the RNA level with age in our IVM‐MII oocytes (Table S5, Figure 2d). On the other hand, the most enriched GO terms for the set of genes presenting lower transcript representation with age were mostly related to mitochondrial function (Table S5, Figure 2d).

In order to assess to which degree overrepresented genes in GV or IVM‐MII oocytes also changed in RNA representation with age, we intersected stage and age markers through Venn diagrams (Figure S4). We observed that only a small proportion of transcripts differentially represented with maturation stage changed with age and that the majority of transcripts changing with age were not maturation stage markers (22% for GV and 3% for IVM‐MII).

Overall, in our data set over 1700 genes changed in transcript representation in correlation with women's age. In general, the majority of transcripts changing with age were not overrepresented transcripts in a specific maturation stage, supporting the idea that ageing does not affect overall cell identity (Wang et al., 2020). Instead, age has an impact on the transcript representation of specific groups of genes related to chromosome segregation and mitochondrial function, which have been implicated in the age‐related oocyte quality decline (Almansa‐Ordonez et al., 2020; Nagaoka et al., 2012).

### 2.3 Upstream master regulators differ between up‐ and downregulated genes

**FIGURE 3:**
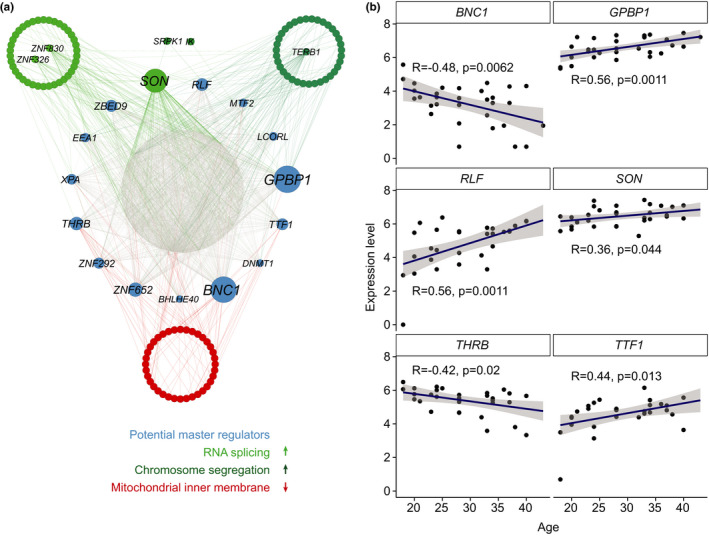
Gene regulatory network analysis. (a) Cytoscape plots from the top 2500 regulatory links among genes found to correlate with age in IVM‐MII oocytes. A list of known human transcription factors was given as an input to Genie3 to be used as potential regulators of the network. In green are genes belonging to two of the GO terms enriched in genes whose RNA levels increase with age, ‘RNA splicing’ (light green) and ‘chromosome segregation’ (dark green). *SRPK1* and *IK* belong to both of these GO terms, and therefore they are plotted in between. In red are genes for which RNA levels decrease with age belonging to the enriched GO term ‘mitochondrial inner membrane’. (b) Expression dynamics of the potential master regulators of genes that correlate with age in IVM‐MII oocytes

IVM‐MII stage is where we found more changes in transcript representation correlated with age and significantly enriched GO terms. In order to identify potential interactions between the transcripts that are altered with age within this maturation stage, we performed a gene regulatory network analysis. As input, we gave an expression matrix including genes whose RNA representation changed with age within the IVM‐MII stage oocytes. Moreover, we used a list of described human transcription factors (Lambert et al., 2018) as potential regulators of the network. When plotting the results (Figure 3a, Table S6) we took into account the terms obtained from the previous GO term analysis (Figure 2d). From the regulatory network analysis, it became clear that the potential upstream master regulators for each of the GO terms were different. We observed GC‐Rich Promoter Binding Protein 1 (*GPBP1*) and RLF zing finger (*RLF*) as potential regulators of genes related to ‘chromosome segregation’ while basonuclin 1 (*BNC1*), thyroid hormone receptor beta (*THRB*) and transcription termination factor 1 (*TTF1*) appeared to be mostly regulating genes related to ‘mitochondrial inner membrane’. Moreover, the DNA and RNA binding protein SON, which is a splicing factor belonging to the ‘RNA splicing’ GO term, also appeared as one of the main regulators of the network. The expression dynamics of the mentioned potential master regulators mostly followed the tendency of the genes within the GO term they regulate. For example, *GBPB1* and *RLF*, showed an increased RNA representation with age, as did the genes present in the GO term ‘chromosome segregation’ (Figure 3b). In the case of the potential upstream master regulators of the GO term ‘mitochondrial inner membrane’ identified within the genes decreasing in transcript representation with age, *BNC1* and *THRB* followed the same dynamics, while *TTF1* behaved the opposite (Figure 3b).

In addition to transcription factors, other groups of proteins such as those involved in RNA stability or RNA processing might also play a role in regulation of gene expression and RNA abundance. For that reason, we decided to perform a network analysis where we allowed all genes of the network (not only transcription factors) to be potential regulators (Figure S5a, Table S6). Interestingly, the main nodes we obtained in this analysis mostly differed from the ones described above, except for *SON* and *RLF*. Among the main regulators, we found in this round of analysis were genes involved in transcriptional regulation (*CLOCK*, *DHX9*, *ZKSCAN5* and *UHRF1*), genes related to RNA regulation (*SON*, *EDC3*), genes related to DNA damage response (*PDCD5*) and genes that regulate centrosome and mitotic spindle integrity (*HAUS7*) or that might influence mitochondrial activity (*VDAC3*). The expression dynamics of the genes in relation to age is shown in Figure S5b.

Altogether we have hereby identified a number of potential master regulators, which could determine the observed RNA representation changes of specific groups of genes in relation to age in IVM‐MII oocytes.

### 2.4 Impact of body mass index (BMI) on human oocyte transcriptome

**FIGURE 4:**
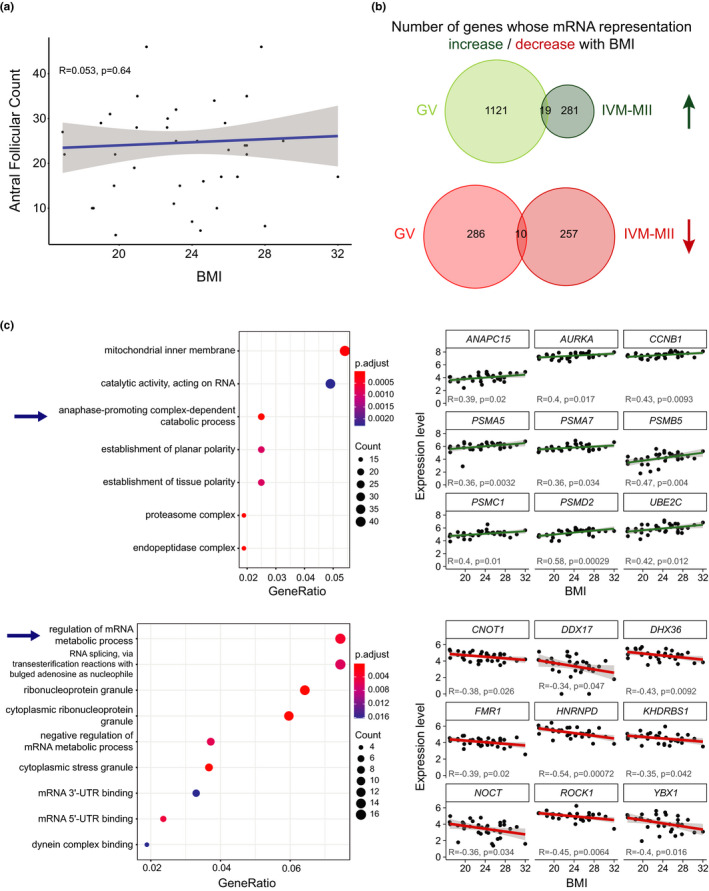
Analysis of gene expression correlation with BMI. (a) Correlation plot between AFC and BMI. (b) Venn diagrams showing the number of genes whose amount of RNA correlates with BMI in GV and IVM‐MII oocytes. In green, genes whose RNA abundance increases with BMI, in red the number of genes whose RNA abundance decreases with BMI. (c) Gene ontology analysis of genes that correlate with BMI in GV oocytes (left) and examples of how expression levels of genes in some of the most significant GO terms (demarcated by arrow) vary with age (right). Upper panel: genes for which expression increases with BMI. Lower panel: genes for which expression decreases with BMI

Both obesity (Machtinger et al., 2012; Shah et al., 2011) and underweight (Brower et al., 2013) in women have been associated with poor oocyte quality and reproductive outcome. Taking advantage of the availability of BMI information from each woman included in our study, we analysed whether this factor could also influence human oocyte quality at the transcriptome level. Our sample set included oocytes from women mostly within the normal [BMI = 18.8–24.9] and overweight [BMI = 25–30] range, plus 1 underweight woman [BMI = 17] and 1 obese woman [BMI = 32]). As we did for age, we considered BMI as a continuous variable and looked at the correlation between BMI and antral follicular count (AFC) as well as BMI and gene expression. Our data set did not show any correlation between BMI and AFC (Figure 4a; *R* = 0.053, *p*‐value = 0.64).

In terms of gene expression, we analysed GV‐ and IVM‐MII‐stage oocytes independently. As opposed to age, BMI influenced transcript representation in GV oocytes more than it did for IVM‐MII oocytes. In GV oocytes, we found a total of 1436 genes whose transcript representation correlated with BMI, mostly positively (79.4%, Figure 4b, Table S7). For IVM‐MII oocytes, a total of 567 genes were found to change in transcript representation with BMI. Approximately half of the genes (300) positively correlated with BMI while the other half (267) correlated negatively. As was the case for RNA representation changes associated with age (Figure 2c), in this instance we observed almost no overlap between genes whose RNA representation changed in GV or IVM‐MII oocytes in relation to BMI (Figure 4b).

Gene ontology analysis revealed no specific term enriched within the lists of genes correlating with BMI in IVM‐MII oocytes. For GV oocytes, the list of genes increasing in transcript representation with rising BMI was enriched in GO terms like ‘mitochondrial inner membrane’, ‘catalytic activity, acting on RNA’, ‘anaphase‐promoting complex‐dependent catabolic process’ and ‘establishment of (planar/tissue) polarity’ among others (Figure 4c, Table S8). For RNAs that decreased in representation with rising BMI in GVs, GO term analysis revealed terms mostly related to regulation of RNA metabolism and RNA splicing.

Overall, BMI affected oocyte transcript levels of specific pathways especially in GV oocytes, which potentially could be related to the decline in oocyte quality and fertility in women with abnormal BMI.

## 3 DISCUSSION

### 3.1 This study in light of previous work

In this study, we have investigated the effect of age on oocyte quality at the transcriptome level. We have used Smart‐seq2 (Picelli et al., 2013) single‐cell RNA sequencing instead of microarrays or bulk RNA‐seq protocols commonly used in previous studies. Moreover, while comparable recent studies analysed only a small number of oocytes (Hendrickson et al., 2017; Reyes et al., 2017; Zhang et al., 2020), our data set includes a large number of single oocytes (*n* = 72) from a large cohort of women, thereby increasing our statistical power in comparison to previous studies.

The design of our study allowed us to compare the transcriptomes of single oocytes obtained at OPU (oocyte pick‐up) time from women of a wide‐ranging age‐span (*n* = 37, 18–43 years) at two stages, GV and in vitro matured (IVM‐MII). Besides the identification of maturation stage‐specific transcripts, our data set allowed us to analyse age‐related oocyte quality decline over time rather than comparing oocytes above or below an arbitrary age threshold. In addition, we used our dataset to identify transcriptomic changes associated with BMI.

### 3.2 Maturation stage is the main differentiator of oocyte transcriptomes

In our analysis, oocyte maturation stage emerged as the main driver of transcriptomic variability. During growth, oocytes are transcriptionally active and once they reach the end of their growth phase (fully grown GVs), oocytes become transcriptionally inactive to complete their nuclear maturation (MIIs) (De La Fuente & Eppig, 2001). In mouse oocytes, it has been observed that during maturation from GV to MII stage, transcripts are degraded at large scale and only transcripts related to pathways essential for the unique MII characteristics are selectively protected from degradation (Su et al., 2007). Hence, it is not surprising that we detected a higher number of genes with increased transcript representation within the GV population (4445) in comparison with IVM‐MII oocytes (324). Furthermore, we observed in GV oocytes GO terms enriched for transcripts related to mitochondrial function and translation (Figure 1f, Table S3). This suggests a degradation of transcripts related to those biological processes during maturation, in line with a reduction of protein synthesis and energy production in IVM‐MII oocytes as previously described in mice (Su et al., 2007).

On the other hand, considering the transcriptional silencing and specific transcript degradation from GV to MII stage, we were surprised to find a number of genes (324) with higher transcript representation in IVM‐MII oocytes than in GVs. A plausible explanation for this result might be related to the poly(A)‐tail length of the detected transcripts. Studies performed in *Drosophila* (Lim et al., 2016), *Xenopus* (Fox et al., 1989) and mouse oocytes (Takei et al., 2020; Yang et al., 2020) have shown a global increase in the poly(A) tail length of mRNAs during oocyte maturation. Some of these mRNAs will keep a long poly(A) tail while others will go through a process of deadenylation at the final oocyte maturation stages (Yang et al., 2020). mRNAs with longer poly(A) tails are preferentially detected in oligo‐dT‐based sequencing libraries (Yang et al., 2020), such as the one used in this study. Therefore, the transcripts we saw overrepresented in the IVM‐MII population in comparison with GVs could be reflecting transcripts that had their poly(A) tail elongated during in vitro maturation. It has been shown in a number of model organisms such as flies, frogs, fish and mice that transcripts with elongated poly(A) tails are more stable and more efficiently translated during oogenesis and encode for proteins important for oocyte maturation and early embryonic development (Lim et al., 2016; Subtelny et al., 2014; Takei et al., 2020; Yang et al., 2020). Consistent with this, we observed in our GO term analysis an enrichment of transcripts related to chromosome condensation in IVM‐MII oocytes, in line with the establishment of metaphase chromosomes at MII stage. Altogether, our data are in concordance with the selective degradation of GV‐specific RNA molecules and also the poly(A) tail elongation and protection from degradation of MII‐specific transcripts as previously described in model organisms. These processes therefore appear to be conserved during oocyte maturation between humans and other species.

### 3.3 Transcripts affected by age are related to oxidative stress, mitochondrial function, chromosome segregation and RNA metabolism pathways

We observed that the transcriptomes of IVM‐MII oocytes were more affected by age than those of GV oocytes, based on the number of genes with increased or decreased RNA representation. This trend has been also previously observed in mouse oocytes (Pan et al., 2008). Furthermore, we observed that the genes whose transcript representation changed with age differed between GV and IVM‐MII oocytes. A similar observation has been made in a study where age‐related changes in the transcriptome of immature non‐human primate oocytes spanning from primordial to antral follicles were assessed (Wang et al., 2020). In that case, genes differentially expressed with age were found to be oocyte growth stage‐specific. We acknowledge the differences in Wang's study and ours regarding the material analysed: in vivo developing immature oocytes from cynomolgus monkeys obtained after whole ovary dissociation vs. human oocytes obtained after an ovarian stimulation protocol and in vitro maturation. Nonetheless, the fact that in both cases ageing affects distinct stages of oocyte development differently is worth noticing.

However, we found it surprising that genes with altered transcript representation with age were rarely overlapping between GVs and IVM‐MIIs, since minimal transcription is taking place between both stages (De La Fuente & Eppig, 2001). In fact, our sampling consists of obtaining GVs from women of different ages and in vitro maturing a part of them to MII oocytes. Therefore, we expected that age‐related altered transcript representation in GVs would be carried along to IVM‐MII oocytes. Part of the discrepancies observed in altered transcript representation between GVs and IVM‐MIIs might stem from the in vitro maturation process itself. More importantly, while age‐related transcriptome alterations in GVs could be transcription‐based, changes in transcript representation in IVM‐MII oocytes would occur mostly on a post‐transcriptional level, thereby affecting different transcripts in IVM‐MIIs than in GVs. This would be consistent with the dramatic transcriptome remodelling by transcript‐specific degradation and polyadenylation pathways during oocyte maturation observed across species (Lim et al., 2016; Su et al., 2007; Subtelny et al., 2014; Yang et al., 2020), which would impact specifically transcripts in MII oocytes if aberrant with age. It is noteworthy that we observed a decrease in the transcript representation for polyadenylation element binding protein *CPEB2* with age in IVM‐MII oocytes. CPEB2 is a potential regulator of cytoplasmic RNA polyadenylation during oogenesis and is important for porcine oocyte meiotic maturation to MII stage and early embryogenesis (Prochazkova et al., 2018). As cytoplasmic polyadenylation is known to regulate stability and translation of maternal‐effect mRNAs for protein production during oogenesis (Susor & Kubelka, 2017) decrease of *CPEB2* transcript with age might be related to the impaired quality of old IVM‐MII oocytes. Indeed, it has been recently shown that altered translation of specific transcripts is a contributing factor to the age‐related quality decline in mouse oocytes (del Llano et al., 2020). Further studies will be needed to assess the role of CPEB2 in human oocytes.

We did not find specific GO terms enriched within the group of genes presenting altered transcript representation with age in GV oocytes. Nevertheless, among the transcripts which decreased in representation in GVs with age, we found the glutathione peroxidase GPX1, which protects cells against oxidative damage. Interestingly, *GPX1* transcript has been shown to decrease with age in immature non‐human primate oocytes contained within primary follicles, although it did not change in oocytes from antral follicles as in our case (Wang et al., 2020). Additionally, we also found PRDX1 to decrease in transcript representation with age within the IVM‐MII population. PRDX1 belongs to the peroxiredoxin family of antioxidant genes, has been suggested to play an antioxidant protective role and to be a putative marker for developmental competence in cow oocytes (Romar et al., 2011). Therefore, we found genes related to oxidative stress protection to decrease in transcript representation with age, in both GV oocytes (*GPX1*) and IVM‐MII oocytes (*PRDX1*). Indeed, with age, an increase of oxidative molecules and a decrease in the expression of oxidation protective genes occurs—a phenomenon known as the ‘oxidative stress theory of ageing’ (Liguori et al., 2018). A decrease in the expression of genes responsible for protecting against oxidative stress has been previously observed as a general feature of ageing in mouse, non‐human primate and human oocytes (Lim & Luderer, 2011; Reyes et al., 2017; Wang et al., 2020), and it is now also reflected in our data set. Altogether, these data indicate that ageing also leads to increased oxidative stress in human oocytes, which might in turn translate into poor oocyte quality.

For IVM‐MII oocytes, gene ontology analysis on transcripts changing with age revealed terms mainly related to chromosome segregation, cell cycle regulation, mitochondrial function and RNA metabolism. All of these biological processes have been previously reported to be altered with age in the human oocyte (Almansa‐Ordonez et al., 2020; Grøndahl et al., 2010; Steuerwald et al., 2007). In fact, if we take a closer look at the entire list of GO terms enriched in the set of genes either with increased or decreased RNA representation with age, we detect both discrepancies and concordances with what has been previously published. For example, ‘DNA repair’ was found to be enriched in old MII human oocytes (Grøndahl et al., 2010), and we also found ‘regulation of DNA repair’ among our GO terms enriched in transcripts increasing in representation with age. Another example is the GO term ‘mitochondrial membrane’, which was also found to be downregulated in older oocytes (Steuerwald et al., 2007). Nonetheless, in the same publication the GO term ‘cell cycle checkpoint’ was found to be downregulated in older oocytes, while we found it in the set of genes with increased RNA representation with age.

Advanced maternal age has been shown to be directly related to an increased rate of aneuploidies, with cohesin loss and centromeric abnormalities being the most studied underlying causes (Burkhardt et al., 2016; Gruhn et al., 2019; Smoak et al., 2016; Tachibana‐Konwalski et al., 2010; Zielinska et al., 2019). Strikingly, in our data, among the top hits of biological processes affected by maternal age in IVM‐MII oocytes were chromosome and chromatid segregation‐related terms (Table S5). Our results therefore point towards the hypothesis that not only protein stability of key chromosomal factors but also abnormal changes at the RNA level of those genes might contribute to the increased aneuploidy rate with advanced maternal age. Indeed, Fragouli and colleagues described a link between transcriptomic alterations of genes involved in biological processes such as spindle assembly or chromosome alignment and aneuploidy of oocytes (Fragouli et al., 2010). However, the increase in transcript representation of the genes belonging to the GO term ‘chromosome segregation’ in our study was very modest, rarely exceeding 50%. This is important to consider as it could suggest a minimal biological effect. Alternatively, this brings us back to the hypothesis of a potential bias in detection of transcripts with long poly(A) tails, which are preferentially retained and translated in oocytes (Yang et al., 2020). This could suggest that abnormal protein levels of chromosomal factors in aged oocytes might trigger compensatory mechanisms including increased polyadenylation of those transcripts for further translation. In support of this hypothesis, a recent study profiling the translatome of ageing mouse oocytes identified transcripts related to meiotic spindle formation and chromosome alignment as misregulated during translation (del Llano et al., 2020).

Taken together, our results point out that transcripts related to chromosome segregation, cell cycle regulation, mitochondrial function and RNA metabolism are altered in human oocytes during ageing. Moreover, it remains to be shown to which degree small changes in transcript representation directly impact oocyte quality or are alternatively a consequence of bigger alterations at the post‐transcriptional level during late stages of oocyte maturation.

### 3.4 Identification of potential master regulators of age‐related changes in oocyte transcriptome by network analysis

In order to identify master regulators of the pathways affected by age, we took a network analysis approach (Huynh‐Thu et al., 2010) using a list of human transcription factors (Lambert et al., 2018) as potential regulators of the network. Among the potential upstream regulators in IVM‐MII‐stage oocytes, we identified the zinc finger transcription factor *BNC1*. It is expressed in germ cells of both ovaries and testes and plays a role in the regulation of rRNA transcription (Zhang et al., 2007). Specifically, *BNC1* is expressed in oocytes present within secondary follicles and in ovulated oocytes and its deficiency has been associated with premature ovarian failure. Furthermore, knock‐down of BNC1 in human oocytes leads to impaired meiotic maturation and a decrease of the oocyte‐derived proteins BMP15 and p‐AKT (Zhang et al., 2018). Considering that we observe BNC1 transcript representation to decrease with age in IVM‐MII oocytes, this could provide a link between ageing and impaired meiotic maturation. Furthermore, in mouse oocytes Bnc1 knock‐down leads to impaired RNA polymerase I and II transcription, altered oocyte morphology and a failure of embryos to develop beyond the 2‐cell stage resulting in female subfertility (Ma et al., 2006).

Another potential master regulator that we identified in our gene regulatory network analysis is *SON*. This gene encodes an RNA binding protein, which promotes pre‐mRNA splicing, specially of transcripts presenting weak splice sites and transcripts related to cell cycle and DNA repair (Lu et al., 2014). Interestingly, *SON* not only belongs to the GO term ‘RNA splicing’, which is found among genes with increased transcript levels with age, but we also identified it as a potential regulator of RNA splicing‐related genes. This is in line with extensive autoregulatory cross‐talk within the splicing machinery (Papasaikas et al., 2015). In a study comparing failed‐to‐mature oocytes with IVM‐MII oocytes transcriptome, RNA splicing was one of the main altered pathways, suggesting that impairments in RNA splicing can constitute a major roadblock for oocyte maturation (Li et al., 2020). Therefore, our results suggest that alterations in the RNA splicing machinery might lead to difficulties in oocyte maturation with ageing, and therefore, contribute to the observed oocyte quality decline.

### 3.5 Transcriptomic changes associated with BMI

In addition to age, BMI is a key driver affecting oocyte quality and female reproductive fitness (Brower et al., 2013; Machtinger et al., 2012; Shah et al., 2011). Among the enriched GO terms for RNAs increasing with BMI in GV oocytes, we found ‘anaphase‐promoting complex‐dependent catabolic process’, which includes the anaphase‐promoting complex subunits *ANAPC11* and *ANAPC15*, as well as *AURKA* (Aurora kinase A), which is important for microtubule nucleation during meiotic spindle assembly in mouse oocytes (Namgoong & Kim, 2018; Saskova et al., 2008; Solc et al., 2012). This could explain previously reported spindle abnormalities in oocytes from obese women (Machtinger et al., 2012).

In addition, RNA splicing and other GO terms related to RNA metabolism/dynamics were not only influenced by age in our dataset, but also by BMI in GV stage oocytes, where we observed a decrease in RNA levels for genes associated with these pathways. An example is the downregulation of CNOT1 (CCR4‐NOT Transcription Complex Subunit 1) with increasing BMI, a scaffolding unit of the CCR4‐NOT complex, which is involved in RNA‐deadenylation, RNA degradation and translational repression (Shirai et al., 2014). The CCR4‐NOT complex has been implicated in the selective deadenylation and degradation of transcripts during meiotic maturation in mouse oocytes and the disruption of the CNOT6L subunit of the CCR4‐NOT complex caused defects in microtubule‐chromosome organization and resulted in meiotic arrest (Sha et al., 2018; Vieux & Clarke, 2018). This indicates that RNA regulation plays an important role at the later stages of oocyte maturation. Alterations in the transcripts related to these pathways may contribute to impaired meiotic maturation resulting in reduced oocyte developmental competence.

### 3.6 Conclusion

In this study, we have shown that age as well as BMI affect key pathways at the RNA level, which are involved in oocyte maturation and function such as chromosome segregation, mitochondria, RNA metabolism and translation. While many age‐related oocyte defects, as for example chromosome segregation errors, have been attributed to low protein turnover of centromeric or cohesin associated factors during meiotic arrest, it has been less appreciated that as well the transcripts related to these pathways are misregulated during oocyte ageing. Maturing oocytes go through a dramatic rewiring of gene expression dynamics, which includes phases of global transcriptional and translational repression and selective RNA polyadenylation and RNA degradation (Clarke, 2018; De La Fuente, 2018). Our results suggest that some of the effects of advanced maternal age as well as abnormal BMI on oocyte quality and developmental competence, could be driven by alterations at the RNA level.

In summary, we provide with this study a high‐resolution analysis of the pathways affected at the RNA level in correlation with age and BMI in GV and IVM‐MII oocytes. Besides advancing our knowledge on the underpinnings of the age‐ and BMI‐related oocyte quality decline, we deliver a rich resource for the field, guiding further investigations and potential diagnostic or therapeutic developments related to oocyte quality.

## 4 EXPERIMENTAL PROCEDURES

### 4.1 Ethical approval

Approval to conduct this study was obtained from the Ethics Committee for Clinical Research (CEIm) of Clinica Eugin before the beginning. All women included in the study gave their written informed consent prior to inclusion.

### 4.2 Study population

A total of 37 women (*n* = 25 oocyte donors and *n* = 12 patients) were prospectively included in the study from January 2018 until June 2019. Inclusion criteria for patients undergoing IVF/ICSI with own oocytes encompassed advanced maternal age and male factor infertility. Patients with medical conditions involving survivors of cancer, chronic infection (HIV, Hepatitis C) or endometriosis stage IV were excluded from the study. All women had a body mass index (BMI) < 33, normal karyotype and no systemic or reproductive conditions, such as endometriosis. Oocytes from only one cycle of ovarian stimulation per woman were included. For single‐oocyte RNA‐seq analysis, 72 oocytes were collected; 40 of them were included in the study as GV, while 32 were included as MII after in vitro maturation (IVM‐MII). The average women age was 28.8 ± 7.7 (range 18–43), with a mean ovarian reserve (measured by antral follicle count; AFC) of 22.1 ± 10.7 (range 4–46). The characteristics of the ovum pick‐up for each participant are shown in Table S1.

### 4.3 Ovarian stimulation, oocyte retrieval and in vitro maturation

Women were stimulated with highly purified urinary hMG (Menopur®, Ferring, Spain) or follitropin alpha (Gonal®, Merck‐Serono, Spain), with daily injections of 150–300 IU (Blazquez et al., 2014). A GnRH antagonist (0.25 mg of Cetrorelix acetate, Cetrotide®, Merck Serono, Spain) was administered daily from day 6 of stimulation (for donors) or from when a follicle of 14 mm or estradiol ≥400 pg/ml was detected (for patients) (Olivennes et al., 1996). In the case of donors, when 3 or more follicles of ≥18 mm of diameter were observed, final oocyte maturation was triggered with 0.2 mg of triptorelin (Decapeptyl®, Ipsen Pharma, Spain). In the case of patients, when 3 follicles of ≥17 mm of diameter were observed, final oocyte maturation was triggered with 250 µg of alpha‐choriogonadotropin (Ovitrelle®, MERCK) or 0.3 mg of triptorelin (Decapeptyl®, Ipsen Pharma, Spain). Oocyte retrieval was performed 36 h later by ultrasound‐guided transvaginal follicular aspiration. Oocytes were denuded 30 min after pick‐up by exposure to 80 IU/ml hyaluronidase (Hyase‐10x, Vitrolife, Sweden) in G‐MOPS medium (Vitrolife, Sweden), followed by gentle pipetting. Once denuded, oocytes were scored for polar body presence and immature GV was either processed immediately as GV or further cultured in vitro in 50 μl of G2‐PLUS (Vitrolife, Sweden) medium in a humidified atmosphere of 6% CO_2_/94% at 37°C for 30 h, when they were checked again for polar body presence and processed.

### 4.4 Single‐cell RNA sequencing

Full‐length single‐cell RNA‐seq libraries were prepared using the Smart‐seq2 protocol (Picelli et al., 2013) with minor modifications. Briefly, oocytes were dezoned with Pronase (Roche Diagnostics, Spain), and individually placed in 2.3 µl of a lysis buffer containing 0.2% Triton‐X100 (T8787, Sigma) and 1 U/µl RNAse inhibitor (N8080119, Applied Biosystem), and stored at −80°C until use. Reverse transcription was performed using SuperScript II (Thermo Fisher Scientific) in the presence of 1 μM oligo‐dT_30_ VN (IDT), 1 μM template‐switching oligonucleotides (QIAGEN) and 1 M betaine. It is important to note that cDNA conversion was performed using oligo‐dTs, which could potentially introduce a bias towards long‐tail mRNAs. The robustness of our data and significance of our results is not compromised by this fact. cDNA was amplified using the KAPA Hifi Hotstart ReadyMix (Kapa Biosystems) and IS PCR primer (IDT), with 20 cycles of amplification. Following purification with Agencourt Ampure XP beads (Beckmann Coulter), product size distribution and quantity were assessed on a Bioanalyzer using a High Sensitivity DNA Kit (Agilent Technologies). A total of 140 pg of the amplified cDNA were fragmented using Nextera XT (Illumina) and amplified with Nextera XT indexes (Illumina). Products of each of the 96‐well plate were pooled and purified twice with Agencourt Ampure XP beads (Beckmann Coulter). Final libraries were quantified and checked for fragment size distribution using a Bioanalyzer High Sensitivity DNA Kit (Agilent Technologies). Pooled sequencing of Nextera libraries was carried out using a HiSeq4000 (Illumina) to an average sequencing depth of >1 million reads per cell. Sequencing was carried out as paired‐end (PE75) reads with library indexes corresponding to cell barcodes (Unique dual indexing).

### 4.5 Data analysis

#### 4.5.1 scRNA‐seq initial processing

Raw sequencing data were obtained from the Smart‐seq2 protocol as described elsewhere (Guillaumet‐Adkins et al., 2017) with minor modifications. Briefly, initial quality check on the FASTA files was carried out with FastQC quality control suite. Samples that reached quality standards were processed to deconvolute reads and assign them to a single cell by demultiplexing according to pool barcodes. PolyT reads were removed. Sequencing reads were mapped with the STAR v2.5.4b RNA aligner (Dobin et al., 2013) with default parameters, and the reference genome was Gencode release 32 (assembly GRCh38.p13). Gene quantification was carried out using UMI to account for amplification biases (allowing an edit distance up to two nucleotides in UMI comparisons). Only unambiguously mapped reads were considered (Guillaumet‐Adkins et al., 2017). Mapped reads were assigned to genes when they overlapped with exons. Only oocytes with ≥1000 UMIs, ≥10,000 reads, and ≤30% mitochondrial transcripts (35 GVs and 31 IVM‐MIIs) were included for further analysis (Figure S1). These metrics were considered jointly to ensure that the discarded cells with high mitochondrial expression were not metabolically active, but instead low quality damaged cells. UMI counts were normalized using Seurat's (Butler et al., 2018; Stuart et al., 2019) SCTransform pipeline, a modelling framework for the normalization and variance stabilization of molecular count data from scRNA‐seq (Hafemeister & Satija, 2019), which finds sharper biological differences and avoids most technical/confounding factors compared with Seurat's standard pipeline.

#### 4.5.2 scRNA‐seq clustering and differential expression analysis of maturation stages

To cluster the oocytes, we (i) performed a principal component analysis (PCA) using the scaled and normalized 3000 most highly variable genes, (ii) used the top 20 principal components (PCs) and the *FindNeighbors* function to create a k‐nearest neighbour graph based on the lower‐dimensional embedding and (iii) clustered the cells with the *FindClusters* function using the default parameters, including the resolution set to 0.8. To visualize the data set, we used a non‐linear dimensionality reduction technique, *t*‐distributed Stochastic Neighbor Embedding (tSNE), on the top 20 PCs and setting the perplexity hyperparameter to 15. To find the cluster markers, in our case corresponding to the maturation stage, the function *FindAllMarkers* was applied, using the ‘MAST’ test (Finak et al., 2015) and setting the parameters min.pct and logfc.threshold to 0 and only.pos to TRUE. This analysis was performed using the Seurat *R* package (Butler et al., 2018; Stuart et al., 2019).

Differentially expressed genes (DEG) presenting an average fold change over 2 between GV and IVM‐MII oocytes and a *p*‐value smaller than 0.01 were considered as significant maturation stage markers. EnhancedVolcano R package (Blighe et al., 2019) was used for the visualization of the gene's significance and log2FC (lnFC values obtained from the function *FindAllMarkers* were transformed to log2FC using the formula log2FC = lnFC/ln(2)), the names of the top 10 markers per each maturation stage are plotted.

#### 4.5.3 Analysis of gene expression correlation with age

Analysis of gene expression correlation with age was performed using pearson correlation by means of the *cor*.*test* function. This analysis was carried out independently for GV stage oocytes and IVM‐MII stage oocytes. Genes presenting a correlation value (*R*) > =|0.3| and a *p*‐value <0.05 were considered significantly correlated with maturation stage. Plots of gene expression levels correlating with age were obtained using the ggplot2 R package (Wickham, 2016).

Venn diagrams used to evaluate how many genes correlating with age were shared between the GV stage oocytes and the IVM‐MII stage oocytes were plotted using the VennDiagram *R* package (Chen & Boutros, 2011). Lists of the genes positively (increasing RNA representation with age) or negatively (decreasing RNA representation with age) correlating with age in GVs and IVM‐MIIs were used as an input. Proportional Venn diagrams to intersect maturation stage markers with genes, for which RNA levels change with age have been generated with the Eulerr *R* package. (Micallef & Rodgers, 2014; Wilkinson, 2012).

#### 4.5.4 Gene ontology analysis

GO term enrichment analysis was performed using the *enrichGO* function from the ClusterProfiler *R* package (Shannon et al., 2003; Yu et al., 2012). The entire list of maturation stage markers (GV and IVM‐MII independently), filtered for a significant *p*‐value <0.01 and a fold change (FC) > |2|, was used as an input. GO terms with an adjusted *p*‐value (FDR method) below 0.05 were considered significant. For GO term enrichment analysis of the genes correlating with age, the function was run four times: genes that positively correlate with age in GVs, genes that negatively correlate with age in GVs and the same for IVM‐MIIs. Again, GO terms with an adjusted *p*‐value lower than 0.05 were considered as significant.

#### 4.5.5 Gene regulatory network analysis

Gene regulatory networks were analysed using the Genie3 *R* package (Huynh‐Thu et al., 2010). The normalized expression matrix of genes changing in expression (positive or negative correlation) with age in IVM‐MII stage oocytes was given as an input. Either a list of described human transcription factors (TF) (Lambert et al., 2018) or all genes changing RNA amount with age were specified in the parameter ‘regulators’ for the Genie3 analysis. The top 2500 regulatory links were next plotted using Cytoscape3 (Shannon et al., 2003). Genes with the highest number of interactions (over 50 for human TF and over 10 for all genes considered as potential regulators) and a betweenness centrality value over 0.5 were considered the main regulators of the network. For visualization, the main nodes (blue) size represent the number of interactions with other nodes. The colour green represents genes belonging to GO terms enriched in the list of genes positively correlating (going up in expression) with age in IVM‐MII oocytes. The red colour indicates the genes belonging to GO terms enriched in the list of genes negatively correlating (going down in expression) with age in IVM‐MII oocytes. Genes with a number of interactions lower than 50 (TF as regulators) or 10 (all genes considered as potential regulators) have a node size equal to 0.

## CONFLICT OF INTEREST

The authors have no conflict of interest to declare.

## AUTHOR CONTRIBUTIONS

M.B., A.M., H.H., R.V. and B.P. conceived the study. M.B. collected and performed IVM on oocytes. P.L. and S.R. performed single‐cell sequencing. S.L., P.N. and M.E. performed Bioinformatic analysis. F.Z. assisted with data analysis. B.P., R.V. and H.H. acquired funding and supervised the project. S.L., M.B. and B.P. wrote the paper with input from the other authors.

### Open Research Badges

This article has earned an Open Data Badge for making publicly available the digitally‐shareable data necessary to reproduce the reported results. The data is available at https://www.ncbi.nlm.nih.gov/geo/query/acc.cgi?acc=GSE158802.

## Supporting information

Fig S1‐S5

Table S1

Table S2

Table S3

Table S4

Table S5

Table S6

Table S7

Table S8

## Data Availability

The data underlying this article have been uploaded to the Gene Expression Omnibus (GEO) with accession number GSE158802: https://www.ncbi.nlm.nih.gov/geo/query/acc.cgi?acc=GSE158802. Furthermore, data can be explored interactively at:https://marcelosuabayes.shinyapps.io/shiny_smartseq2/.
